# RAI16 maintains intestinal homeostasis and inhibits NLRP3‐dependent IL‐18/CXCL16‐induced colitis and the progression of colitis‐associated colorectal cancer

**DOI:** 10.1002/ctm2.993

**Published:** 2022-08-28

**Authors:** Wen Wang, Cui‐Ling Ding, Meng‐Xue Wu, Wen Guo, Ran Hu, Yan Liu, Zhong‐Tian Qi, Xin‐Ming Jia

**Affiliations:** ^1^ Clinical Medicine Scientific and Technical Innovation Center, Shanghai Tenth People's Hospital Tongji University School of Medicine Shanghai China; ^2^ Department of Biodefence Naval Medical University Shanghai China

Dear editor,

The pathogenesis of inflammatory bowel disease (IBD) and the tumourigenesis of colitis‐ associated colorectal cancer (CAC) are still unclear.^1^ Previously, our group demonstrated that RAI16 deficiency exacerbated dextran sodium sulphate (DSS)‐induced colitis and CAC.^2^ However, the mechanism of RAI16 in regulation of inflammation or tumourigenesis remains unclear. In this study, we showed that RAI16 interacts with dynein cytoplasmic 2 heavy chain 1 (DynC2H1), regulating NOD‐like receptor thermal protein domain associated protein 3 (NLRP3)‐dependent interleukin 18 (IL‐18) maturation in the gut, which leads to exacerbated colitis during DSS treatment. Increased IL‐18 production induces C‐X‐C Motif Chemokine Ligand 16 (CXCL16) secretion, which recruits immunosuppressive myeloid‐derived suppressor cells (MDSCs) and enhances tumour cell proliferation and migration directly, thus, leading to CAC tumourigenesis. The new aspect of the current study is the production of IL‐18 that enhances colitis on the one hand and leads to CXCL16 secretion that enhances tumour proliferation and migration.

Studies have demonstrated the important roles of the gut microbiota and their metabolites in IBD pathogenesis.^3^ WT mice cohoused with RAI16^–/–^ mice showed increased colitis, meanwhile, RAI16^–/–^ mice cohoused with WT mice showed decreased colitis (Figure 1A–D). Under treatment of antibiotic cocktail (ABx), RAI16^–/–^ mice just develop mild colitis by 2.5% DSS treatment similar with WT mice (Figure 1E and F). Transplant of faecal material of RAI16^–/–^ mice by gavage led to more severe colitis (Figure 1E–G), suggesting that gut dysbiosis may contribute to the higher susceptibility of RAI16^–/–^ mice to DSS‐induced colitis. Furthermore, significant increase of *Bacterroidase* and *Prevotellaceae* and decrease of *Clostridia* were noted in stools of RAI16^–/–^ mice compared with those of WT mice (Figure 1I and J). Under DSS treatment, *Prevotellaceae* in colon of RAI16^–/–^ mice is higher than that of WT mice (Figure 1K). RAI16^–/–^ mice colocated with *Prevotella spp*. group is with more weight loss, higher DAI score and higher histological score (Figure 1L–N). These results indicate RAI16 may play roles in maintaining the balance of gut microbiota, and its loss caused the gut dysbiosis, characterised with increase abundance of *Prevotellaceae*, which exacerbated the severity of 2.5% DSS‐induced colitis in RAI16 deficient mice. Consistent with our study, Strowing et al. reported that *Prevotella* colonisation resulted in metabolic changes in the microbiota, which consequently exacerbate intestinal inflammation and potential systemic autoimmunity.^4^

**FIGURE 1:**
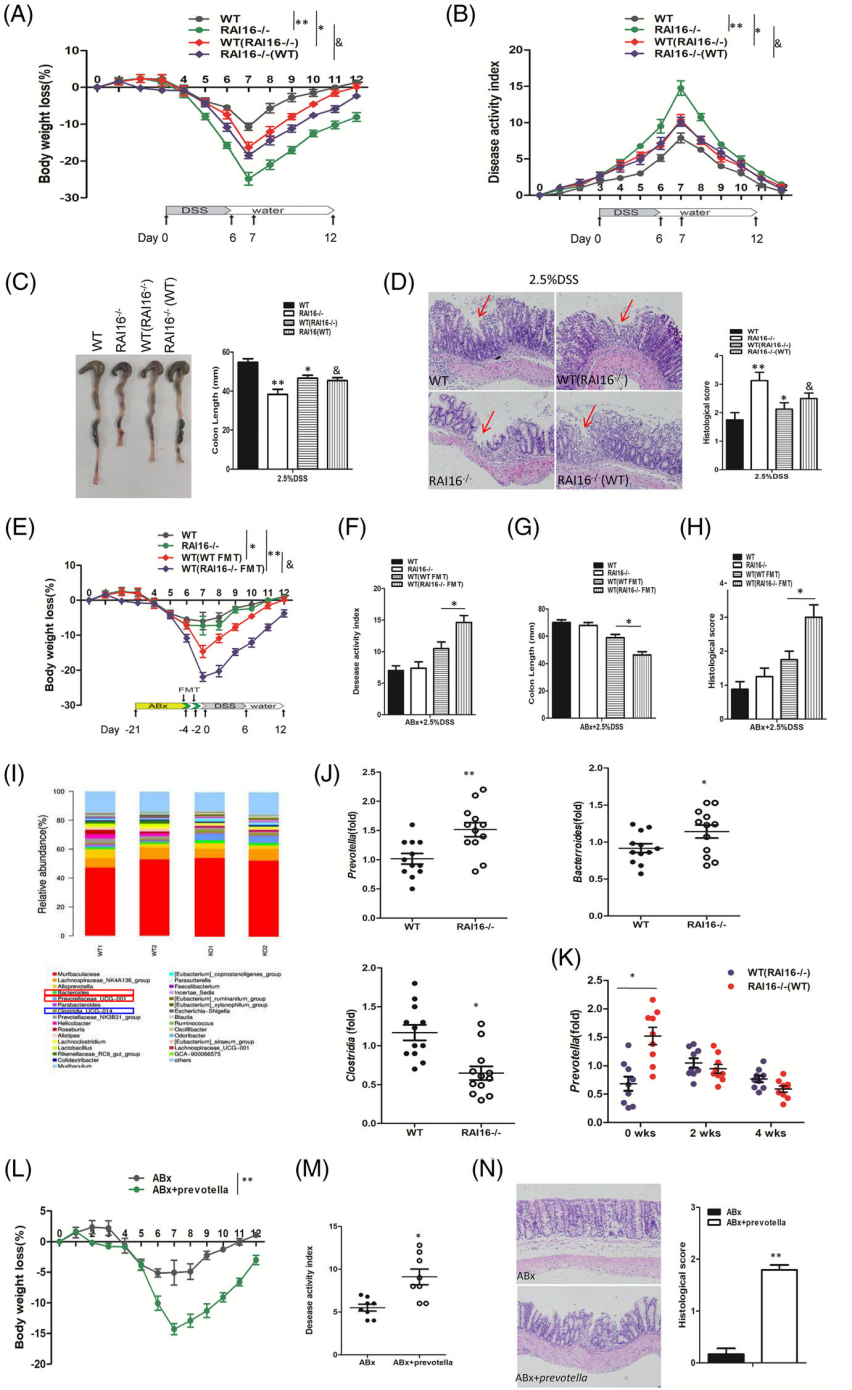
Transferrable dysbiosis in RAI16^–/–^ mice is responsible for exacerbated colitis. (A–D) Separately housed RAI16^–/–^ mice, WT mice and co‐housed WT (WT(RAI16^–/–^) mice or RAI16^–/–^ (WT) mice were administrated of 2.5% DSS for 6 days. Body weight loss (A) and disease activity index (B) of separately housed RAI16^–/–^, WT, co‐housed WT (WT(RAI16^–/–^) and RAI16^–/–^ (WT) mice were monitored daily, colon length (C) and histopathology (D) were determined at day 7 post DSS treatment. (E, H) Pretreated for 3 weeks with an antibiotics cocktail (ABx: ampicillin, kanamycin, vancomycin and metronidazole), then recolonised with either WT or RAI16^–/–^ faecal material by oral gavage, mice were administrated of 2.5% DSS as usual. Body weight loss (E), disease activity index (F), colon length (G) and histopathology (H) were determined as before. (I) Bacterial 16S rRNA‐based analysis of the faecal microbiota from WT and RAI16^–/–^ mice. (J) The indicated key microbial species were tested by quantitative PCR (WT and RAI16^–/–^ mice, *n* = 8–12). The increased abundance of *Prevotella* and *Bacterroidase* and the decreased abundance of *Clostridia* were shown. (K) Relative abundance of *Prevotella* DNA at 0, 2 and 4 weeks of co‐housing was measured by quantitative PCR. (L, N) Pretreated for 3 weeks with ABx as before, then recolonised with *Prevotella spp*. by oral gavage or not, mice were administrated of 2.5% DSS as usual. Body weight loss (L), disease activity index (M) and histopathology (N) were determined as before.

Dysfunction of gut barrier is related to the incidence and severity of DSS‐induced colitis.^5^ Epithelial permeability of RAI16^–/–^ colon is significantly higher than that of WT colon (Figure 2A), indicating a leaky gut in RAI16^–/–^ colon, also suggesting an important role of RAI16 in gut barrier. However, mucosa‐producing goblet cells are even more robust and mucus is intact in RAI16^–/–^ colon (Figure 2B), indicating RAI16 deficiency does not affect goblet cells proliferation and secret mucus. The expression of E‐cadherin, ZO‐1, E‐cadherin, β‐catenin and Claudin‐1 did not show any difference between WT and RAI16^–/–^ IECs (Figure 2C and D), indicating that RAI16 does not affect IECs integrity. In addition, the levels of regenerating islet‐derived protein 3β(Reg3β) and Reg3γ (antimicrobial peptides) were reduced in RAI16^–/–^ IECs (Figure 2E), which may be the reason of dysbiosis and higher susceptibility to colitis of RAI16^–/–^ mice. Furthermore, query BioGrid database (http://thebiogrid.org/122273) DynC2H1 may interact with RAI16 (Fam160B2, Figure 2F). The interaction of RAI16 and DynC2H1 was verified exogenously and endogenously by co‐immunoprecipitation (Figure 2G and H). Furthermore, in RAI16^–/–^ IECs, the interaction of NLRP3 and DynC2H1 was enhanced under DSS treatment (Figure 2I). Caspase‐1 and IL‐18 were significantly increased at 24 h post DSS treatment in RAI16^–/–^ IECs compared with that of WT IECs (Figure 2J). Together, it is suggested that RAI16 interaction with DynC2H1 may be involved in NLRP3 inflammasome‐dependent IL‐18 activation. IL‐18 plays multifaceted roles in colitis, either the lack or excess of IL‐18 were reported to promote colitis.^6^, ^7^

**FIGURE 2:**
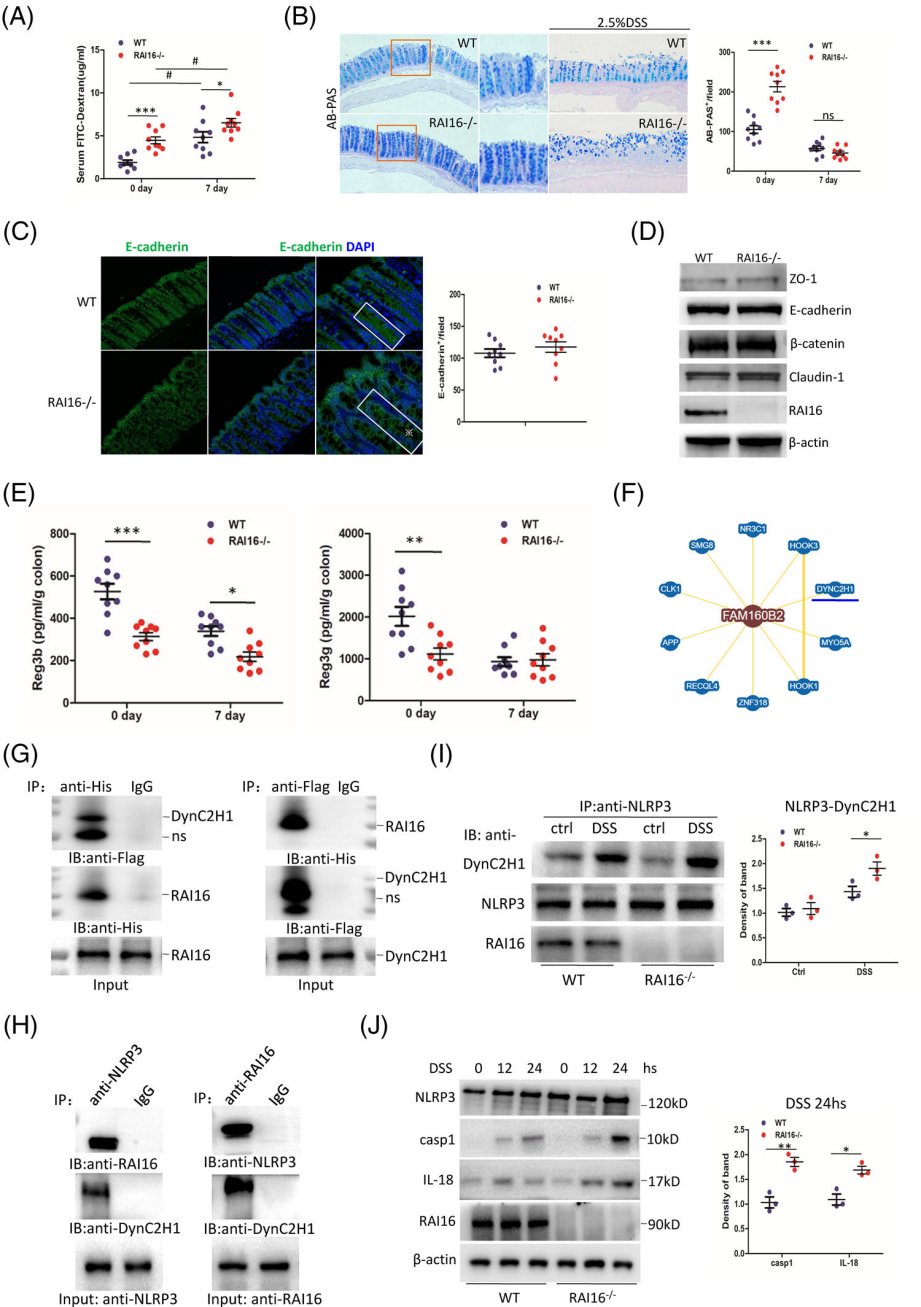
RAI16 interacts with DynC2H1 regulating DSS‐induced NLRP3 activation. (A) Intestinal permeability of WT and RAI16^–/–^ mice (*n* = 9) was determined with FITC‐dextran in vivo. (B) Alcianblue‐Periodic acid Schiff (AB‐PAS; goblet cells) staining of the colons derived from WT and RAI16^–/–^ mice (*n* = 9) treated with or without 2.5% DSS for 6 days and quantitation results are shown in the right. (C) Representative E‐cadherin staining in colon sections derived from WT and RAI16^–/–^ mice (*n* = 9) and quantitation results are shown in the right. (D) Immunoblotting analysis of the expression of selected adherent junction (AJ) and tight junction (TJ) proteins in colonic epithelial scrapings obtained from WT and RAI16^–/–^ mice. (E) Reg3β and Reg3γ levels from WT and RAI16^–/–^ colon tissue explants were measured by ELISA. (F) The predict interaction of RAI16 (Fam160B2) and DynC2H1. (G) HEK293T cells were co‐transfected with RAI16‐His and DynC2H1‐Flag expression vectors. The cell lysates were immunoprecipitated using anti‐Flag or anti‐His and immunoblotted with anti‐His or anti‐Flag. (H) Endogenous co‐immunoprecipitation assay was performed in IECs with anti‐NLRP3 or anti‐ RAI16, and detected with indicated antibodies. (I) IECs treated with DSS (1.0%) or not for 24 h were immunoprecipitated with anti‐DynC2H1, anti‐RAI16 or IgG, and detected with indicated antibodies. (J) IECs from WT and RAI16^–/–^ mice were treated with DSS and immunoblotted with indicated antibodies.

Higher IL‐18, lower TNF‐α and IL‐6 levels were found in colon explants from RAI16^–/–^ mice compared with those from WT mice (Figure 3A). Consistent with the above findings, IL‐18 protein was increased in DSS‐treated RAI16^–/–^ colon tissues by immunoblot (Figure 3B). The increase of IL‐18 was confirmed in RAI16^–/–^ IECs at day 7 after DSS administration (Figure 3C). These results suggest an important role of IL‐18 in RAI16 deficiency‐induced high sensitivity to colitis. Anti‐IL‐18 treatment markedly improved the colitis (Figure 3D–H) and rescued RAI16^–/–^ from AOM/DSS‐induced severe CRC (Figure 3I–K). These data indicated that the severe colitis and associated CAC in RAI16^–/–^ mice depends primarily on elevated production of IL‐18.

**FIGURE 3:**
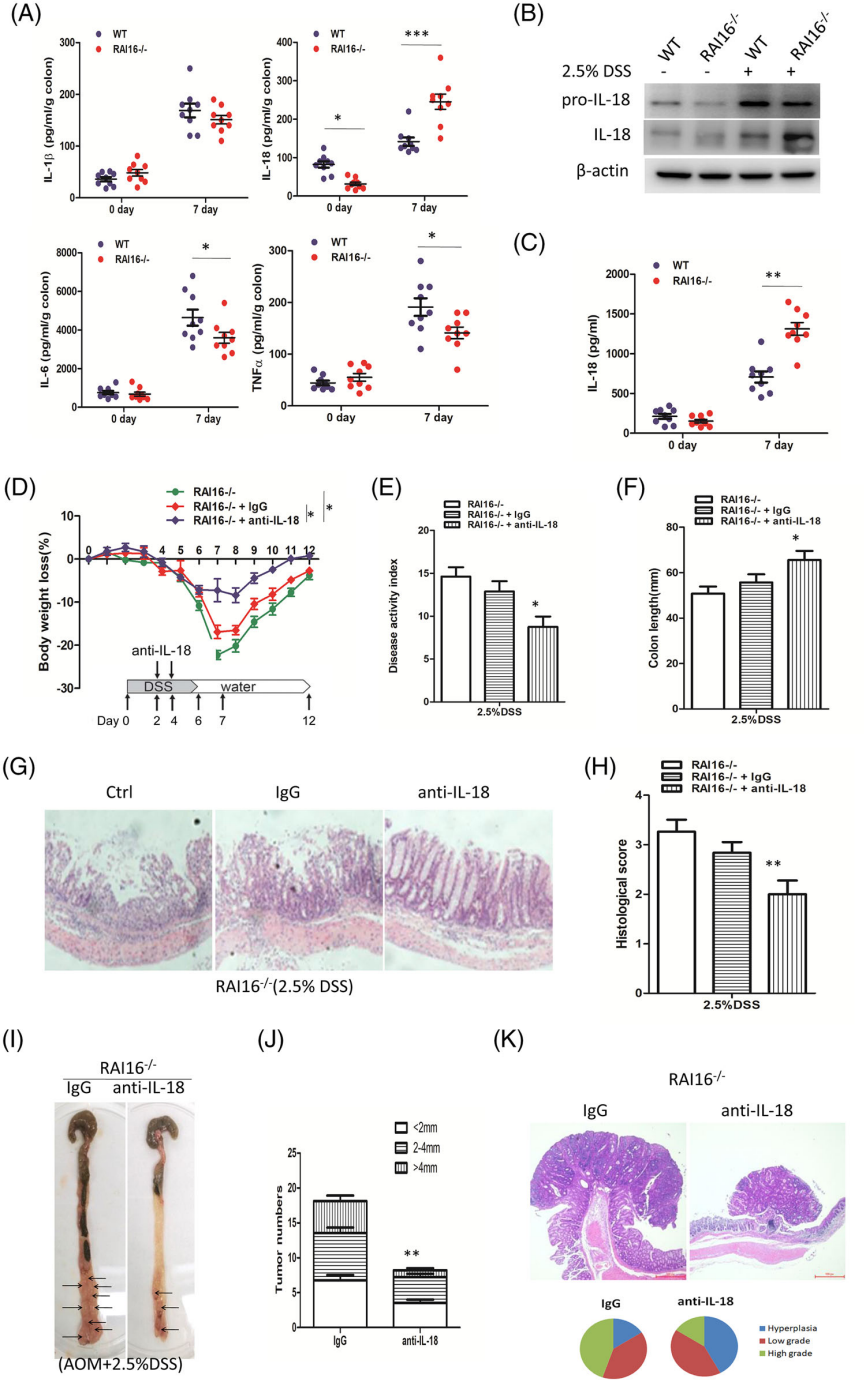
Colonic IL‐18 activation is involved in RAI16 deficiency‐induced colitis and associated CAC progression. (A) IL‐1β, IL‐18, IL‐6 and TNF‐α levels were measured in colon explants from WT and RAI16^–/–^ mice with or without DSS treatment by ELISA. (B) The procession of IL‐18 was determined in colon tissues from WT and RAI16^–/–^ mice with or without DSS treatment by immunoblot. (C) IL‐18 level was measured in supernatant of WT and RAI16^–/–^ IECs with or without DSS treatment by ELISA. (D–H) Mice was intraperitoneally injected a neutralising IL‐18 antibody (anti‐IL‐18,) or control antibody (IgG) every 2 days for 3 times during DSS treatment. Body weight loss (D), disease activity index (E), colon length (F) and histopathology (G) were determined as before. (I–O) Mice were intraperitoneally injected a neutralising IL‐18 antibody (anti‐IL‐18) or control antibody (IgG) once a week for 14 weeks. Tumour number (I), tumour size (J) and tumour grade (K) were analysed on day 100.

By using multi‐chemokine array (AAM‐CHE‐1, Raybiotech, USA), CXCL16, CX3CL1, CXCL1, CCL22 and CCL20 were found to be significantly increased in RAI16^–/–^ TAMs compared with WT TAMs (Figure 4A). The mRNA expressions and secretion of CXCL16 showed the significant increase in TAMs of RAI16^–/–^ mice (Figure 4B and C). Under DSS treatment, CXCL16 secretion in colon tissues of RAI16^–/–^ mice is higher than that of WT mice (Figure 4D). IL‐18 was reported to positively regulate CXCL16 transcription and CXCL16‐dependent aortic smooth muscle cells (ASMC) proliferation.^8^ These results indicated that IL‐18/CXCL16 may be involved in the severe tumourigenesis by RAI16 deficiency. On the other hand, it was found that the percentage of MDSCs (Gr1+CD11b+) in tumour tissues of RAI16^–/–^ mice is much higher than those in tumour tissues of WT mice (Figure 4E). In addition, G‐MDSCs are the major part in these increased MDSCs (Figure 4F). The mRNA expression of Arg1 in distal colons from tumour‐bearing RAI16^–/–^ mice was increased compared with that of WT mice (Figure 4G). These results indicated that RAI16 deficiency may induce immunosuppressive tumour microenvironment characterised with G‐MDSCs infiltration. By migration assay, we demonstrated that RAI16^–/–^ TAMs or recombinant mouse CXCL16 (rmCXCL16, 10 ng/ml) treatment could recruit much more MDSCs (Figure 4H). Moreover, TAM‐ s or rmCXCL16 treatment could enhance the proliferation and migration of HCT116 cells (Figure 4I and J). Neutralising mouse CXCL16 antibody (anti‐mCXCL16, 300 ng) treatment for 14 weeks reduced MDSCs (Gr1+CD11b+) or G‐MDSCs (Gr1+CD11b+Ly6G+) (Figure 4K and L) in tumour tissues. As expected, tumour number, tumour size and tumour grade from anti‐mCXCL16‐treated RAI16^–/–^ mice were decreased (Figure 4M–O). These results indicated that CXCL16 was involved in RAI16 deficiency‐induced severe tumourigenesis by recruiting MDSCs and enhancing tumour cell proliferation and migration together. It has been reported that TAM CXCL16 increases migration and proliferation of cancer cells and recruited MDSCs into the tumour nest.^9^, ^10^ Our findings of enhancement of tumour cell proliferation and migration and recruit of MDSCs by CXCL16 strengthens the knowledge of CXC chemokine in tumourigenesis.

**FIGURE 4:**
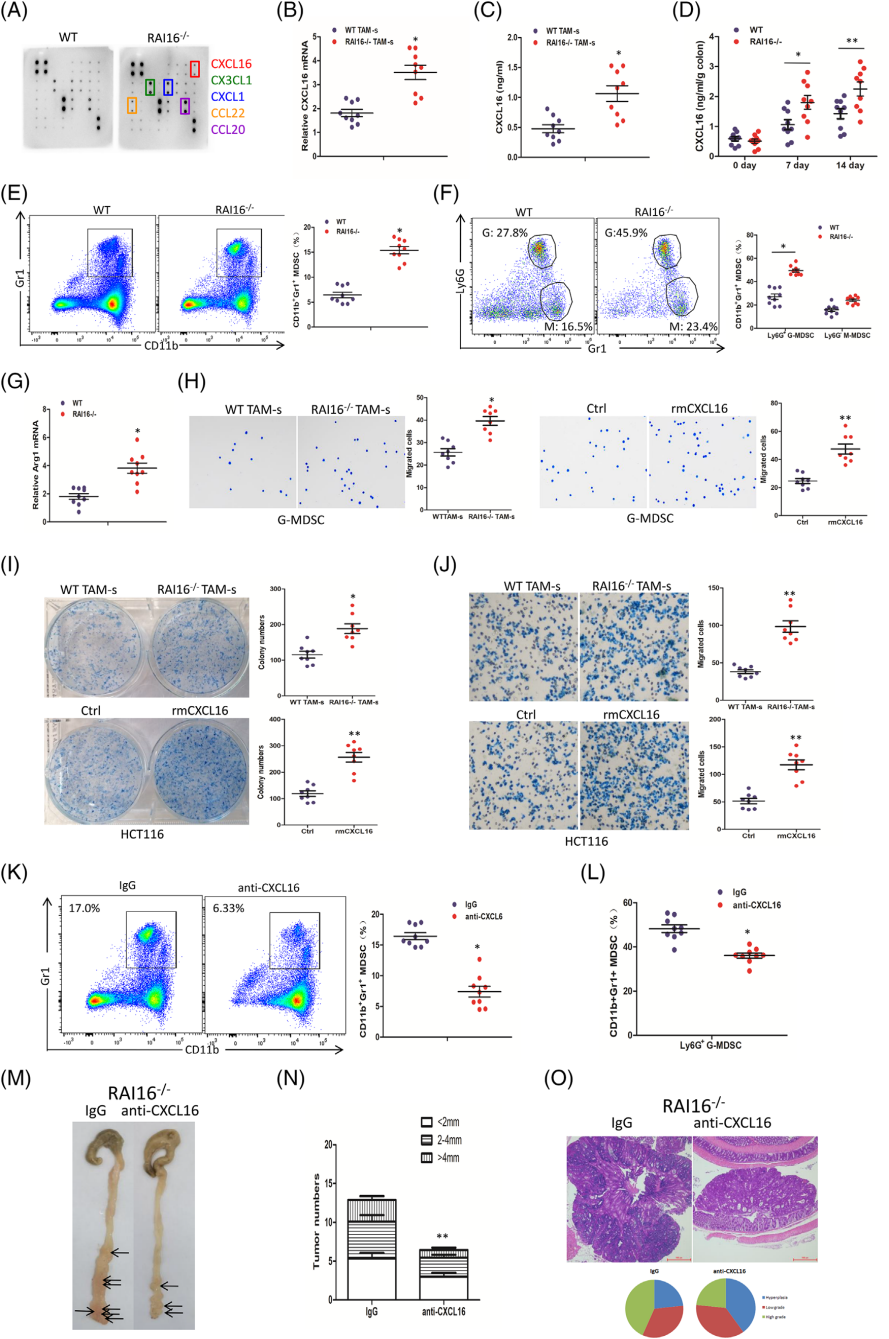
RAI16 deficiency increases CXCL16 production, which recruits immunosuppressive MDSCs and enhances tumour cell proliferation and migration. (A) Multi‐chemokine array proteome profiler (AAM‐CHE‐1, Raybiotech) of supernatants from primary TAMs derived from WT or RAI16^–/–^ tumours. (B) Relative mRNA expression of CXCL16 in TAMs from tumours of WT or RAI16^–/–^ mice was measured by qRT‐PCR. (C) The expression of CXCL16 by TAMs of WT or RAI16^–/–^ mice was measured by ELISA. (D) The expression of CXCL16 in colon tissues WT or RAI16^–/–^ mice with or without DSS treatment. (E) Gr1^+^CD11b^+^ MDSCs in tumour tissues of WT and RAI16^–/–^ mice were assessed by flow cytometry. (F) Gr1^+^Ly6G^+^ G‐MDSCs and Gr1^+^Ly6G^–^ M‐MDSC were further assessed from CD11b^+^ MDSCs by flow cytometry. (G) Lysates of distal colons from tumour‐bearing WT and RAI16^–/–^ mice were prepared, and Arg1 mRNA expression was analysed using qRT‐PCR. (H) Transwell migration assay was performed in MDSCs by TAM culture supernatant (TAM‐s) or recombinant mouse CXCL16 (rmCXCL16, 10 ng/ml) treatment. (I, J) Colony formation assay (I) and Transwell migration assay (J) were performed in HCT116 cells by TAM‐s or rmCXCL16 (10 ng/ml) treatment. K‐M. Mice was intraperitoneally injected a neutralising CXCL16 antibody (anti‐CXCL16) or control antibody (IgG) once a week for 14 weeks. Gr1^+^CD11b^+^ MDSCs (K) or Gr1^+^Ly6G^+^ G‐MDSCs and Gr1^+^Ly6G^–^ M‐MDSCs (L) in tumour tissues from anti‐CXCL16 and IgG‐treated RAI16^–/–^ mice were assessed by flow cytometry. Tumour number (M), tumour size (N) and tumour grade (O) were analysed on day 100

This study depicts the roles of RAI16 and how loss of RAI16 promotes colitis and CAC (Figure S1). According to public data sets, RAI16 mRNA expression was reduced in IBD patients and human CRC tissues and associated with tumour metastasis and overall survival (Figure S2), suggesting a causal link between RAI16 reduction and IBD and CRC pathogenesis. In a conclusion, our study revealed that RAI16 interacts with DynC2H1 regulating NLRP3‐dependent IL‐ 18/CXCL16 signalling, which contribute to colitis and associated CAC progression.

## CONFLICT OF INTEREST

The authors declare that they have no competing interest.

## Supporting information

Supporting Material

Supporting Material

Supporting Materiali

Supporting Material
