# Supplementary material for: RAI16 maintains intestinal homeostasis and inhibits NLRP3‐dependent IL‐18/CXCL16‐induced colitis and the progression of colitis‐associated colorectal cancer
Source: Clin Transl Med. 2022 Aug 28;12(8):e993. doi: 10.1002/ctm2.993 (PMC9420419; doi:10.1002/ctm2.993)
Supplement: Supplementary file 1 — Supporting Material [file CTM2-12-e993-s001.pdf]

SUPPLEMENTARY MATERIALS AND METHODS

Mice

The heterozygous pairs of RAI16$^{-/-}$ mice were used to generate homozygous RAI16$^{-/-}$ and littermate wild type (WT) mice for experimental studies. All animals were maintained by the Laboratory Animal Care Center of Tongji University and provided chow and water adlibitum. All experiment procedures were approved by the Institutional Animal Care and Use Committee of Tongji University and all experiments were performed on age- and gender-matched, randomly assigned mice in accordance with relevant guidelines and regulations.

DSS induced ulcerative colitis and AOM-DSS induced CAC model

RAI16$^{-/-}$mice and wild type (WT) littermate mice (6-7wk of age, bodyweight: 20-22g) were given 2.5% DSS (wt/vol, MP Bio) for 6 days and then regular sterile water for 6 days. RAI16$^{-/-}$ mice and WT littermate mice in control groups were given regular sterile water for all 12 days. On the 7th or 12thday, all mice were sacrificed for further analysis. For induce CAC model, RAI16$^{-/-}$ and WT mice were injected intraperitoneally with AOM (Sigma-Aldrich) at 10 mg/kg body weight. Five days later, mice were given three cycles of 6 days 2.5% DSS and 14 days regular sterile water. The body weight loss of these mice was monitored daily, and the mice with >30% body weight loss were considered dead and killed. After completion of the whole AOM-DSS regimen, these mice were sacrificed (at day91), colons were removed and cut longitudinally. The number and size of tumors in colon of each mouse were blindly counted and measured.

Antibiotic treatment and fecal microbiota transplant

For the antibiotics treatment, mice were equally treated with a combination of antibiotics (vancomycin, 1mg/mL; ampicillin, 1mg/mL; kanamycin, 1mg/mL and metronidazole, 1mg/mL; Sigma-Aldrich) for 3 weeks in the drinking water. For fecal microbiota transplant (FMT), mice were treated with antibiotics as described above and then received fecal content from WT or RAI16$^{-/-}$ mice. Briefly, donor animals were weight-, gender- and age-matched single-housed WT or RAI16$^{-/-}$ mice. Mice were killed, and the cecal content was immediately diluted in sterile phosphate-buffered saline, filtered through a 500μm mesh and administered equally to each

group of mice. Recipient mice were given 48h to recover before DSS administration.

Isolation of fecal DNA and 16S rDNA sequencing

Fecal samples were freshly collected from colons, frozen in liquid nitrogen and stored at -80°C until further processing. DNA in colonic fecal samples was extracted using the QiaAmp DNA Stool Kit (Qiagen, Valencia, USA) according to the manufacturer's instructions and quantified on a Synergy HT Multimode plate reader (BioTek, Winooski, USA). High-throughput was performed in Hiseq 2500 platform (Illumina) with Paired-End sequencing method (PE250)by Oebiotech (Shanghai, China). In brief, the 16S rRNA gene with V4 regions was amplified with F515/R806 primers (GTGCCAGCMGCCGCGGTAA and GGACTACHVGGGTWTCTAAT). TruSeq® DNA PCR Free Sample Preparation Kit was used to construct the amplicons libraries. The data retrieved was assembled and screened by Oebiotech (Shanghai, China). The statistically gut microbial community composition differences and diversity indices between the samples of RAI16$^{-/-}$ and WT mice were computed nonparametric unpaired t-test ($P<0.05$) by using GraphPad Prism 7.

Quantitative real-time PCR

DNA in colonic fecal samples was extracted as above. The primer pairs for 16S rDNA genes were showed as Table S1. Total RNA was isolated using TRIzol reagent (Invitrogen), treated with DNase I, reverse transcribed with iScript (Bio-Rad, Hercules, USA) and analyzed by iTaq Universal SYBR Green real time PCR assays (Bio-Rad). The SYBR Green primer pairs for cytokines including Reg3b, Reg3g, IL-1β, IL-6, IL-18, Arg1 and Gapdh were used (2). The cycling parameters were as follows: 95°C for 15s and then 55.0°C for a total of 40 cycles. For each primer set, the quality of the melting curve was checked to ensure for the specificity of the amplification.

Bacteria preparation

Prevotella spp. (ATCC BAA-1639) was cultured in an anaerobic condition according to ATCC culturing guidelines. Bacteria were then washed and resuspended in HEPES buffer for infection by oral gavage. Mice pretreated with antibiotics as described above were orally garages with 6×10^{7} *Prevotella spp.* for 5 days.

Determination of disease activity index (DAI)

Weight loss, stool consistency and any presence of occult or macroscopic blood were determined daily until mice were killed at days 0, 7 or 12. Stool consistency and rectal bleeding were analyzed as previously described (2).

Histology

Mice were killed, and entire colons were isolated, measured and opened along the mesenteric border. Mesenteric fat and fecal material were removed, and colons were aseptically flushed several times with Hank's balanced salt solution (HBSS). The colon was fixed in 10% formalin overnight, embedded in paraffin, sectioned and stained with hematoxylin and eosin (H&E) by ServiceBio (Wuhan, China). The severity of colitis was scored histologically as previously described (2).

Colon tissue explants

Whole colonic tissue from WT or RAI16$^{-/-}$ mice were cut in longitudinally and washed extensively to remove feces. Biological triplicates of 1cm colon pieces per mouse were isolated; preincubated in HBSS supplemented with 100μg/ml gentamicin for 30min; rinsed and cultured in complete RPMI-1640 medium supplemented with 10% FBS, 1% penicillin-streptomycin 100μg/ml gentamicin and 10 mM HEPES. Supernatants were collected after 18 h, cleared by centrifugation at 12,000g for 10 min at 4°C and stored at -80°C until use.

Isolation of tumor associated macrophages (TAMs)

Single cell suspensions were prepared from fresh tumors using Tumor Dissociation Kit (Miltenyi Biotec GmbH). The suspension was filtered through a 70 um stainless steel wire mesh to generate a single-cell suspension. The suspension was centrifuged and washed twice with PBS. Cells were left to adhere in serum-free RPMI 1640 for 40 min. Non-adherent cells were washed away. All operations were performed according to the manufacturer's protocol. Immunofluorescence staining for macrophage markers (CD68) confirmed the identity of the adherent populations. For TAM-conditioned medium collection, the cells remained in serumfree conditions for 24 h prior to medium collection.

Intestinal epithelial cells (IECs) isolation

For culture purpose, the colons from WT or RAI16$^{-/-}$mice were cut into pieces and washed by DMEM for two times, then incubated with digestion buffer (DMEM with collagenase type I and Dispase II) for 2 h in 37 °C. After the incubation, the cell suspension was passed through 100μm cell strainers. After washes, the cells were plated in dishes coated with rat tail tendon collagen type I overnight and were cultured in DMEM with 10% FBS and 1% penicillin/streptomycin. The purities of colonic epithelial cell were confirmed by FACS analysis of EpCAM staining.

Cytokine measurement

IL-1β, TNF-α, IL-6, IFN-γ, IL-18, IL-22, IL-10, IL-12p40, IL-12p70, IL-17A and IL-23 were determined with ELISA kits (Thermo Scientific, USA) from clarified culture supernatants, colon tissue explants or colon lysates. ELISA results were colon weight-adjusted.

Epithelial permeability assay

Barrier function was assessed using the permeability tracer FITC-labeled dextran. At 0 day or 6 days after DSS treatment, WT andRAI16$^{-/-}$ mice were deprived of food and water for 4 h and then gavaged with 40kDa FITC-labeled dextran (Sigma-Aldrich) at 0.6 mg per kg body weight. Blood was retro-orbitally collected after 4 h, and the fluorescence intensity in the serum was measured (excitation: 492 nm; emission: 525 nm). The FITC-dextran concentrations were determined from a standard curve generated by serial dilutions of FITC-dextran.

Cell staining and flow cytometry analysis

Single cell suspensions of tumor tissues were subjected to flow cytometry analyses. For gating strategy, first FSC/SSC was applied to gate the live cells and then according to fluorochrome to make the subsequent gates. All the samples in the same experiments and comparisons were gated under the same parameters. To detect MDSC, cells were stained with living dye as well as CD4-FITC, CD11b-APC, Gr1-FITC andLy6G-PE antibodies (eBioscience, 1:400) on ice for 20 min. After washing with PBS+2% FBS, cells were filtered into flow tubes and analyzed by BD FACSreverse(BD Biosciences), and the data were analyzed with FlowJo (Tree Star).

Plasmid construction and cell transfection

The plasmid of pLenti6-RAI16-His was prepared as described previously (2). DynC2H1 (NM_001364519.1) was amplified from mouse colonic cDNA and cloned into pcDNA6.1 vector to generate the Flag-tagged DynC2H1 (pcDNA6.1-DynC2H1-Flag). Transient transfection of 293T cells was performed using Lipofectamine 3000 (Invitrogen) according to the manufacturer's instructions. The expressions of RAI16 and DynC2H1 were confirmed by immunoblot with anti-His or anti-Flag antibodies.

Protein immunoprecipitation

BMDMs were cultured overnight in 1% FEB in DMEM with pen/strep and treated or not with 1.0% DSS for indicated time. Cell lysis was performed using NP-40 lysis buffer containing 50 mM Tris pH 8.0, 150 mM NaCl, 1% NP-40, EDTA-free protease inhibitor cocktail (Roche), 2mM sodium fluoride(Sigma), 10mM glycerophosphate(Sigma) and 1mM sodium orthovanadate (Sigma). Lysates were then incubated with 25 μl of Protein A/G beads and 1μg of anti-NLRP3 (1:100, Proteintech, China) or anti-RAI16 (1:100, Abclonal, China) overnight at 4 °C. Protein A/G agarose was incubated with cell lysates in the absence of an antibody to control for non-specific binding. The immunocomplexes were washed five times with NP-40 lysis buffer, denatured using 2× loading buffer, then subject to immunoblot assay.

Immunoblot analysis

Whole colonic tissue from WT or RAI16$^{-/-}$ mice were cut in longitudinally and washed extensively to remove feces, then suspended in RIPA lysis buffer (Biotime, China) and homogenized. The homogenates were left on ice for 25 min and centrifuged at 14,000 rpm for 15 min at 4°C. The supernatants were collected and stored at -80°C until use. Protein estimations were performed using the BCA protein assay kit (Biotime, China) according to the manufacturer's protocol. The cell lysates were separated by SDS-PAGE, transferred to polyvinylidene difluoride membranes, immunoblotted with the indicated antibodies and visualized with by the Gene Image Capture System (Syngene, Frederick, USA) and quantified using Gene tools (Syngene).

Statistical analysis

Graphs represent the mean±SD. A standard two-tailed unpaired t-test and unpaired, non-parametric Mann-Whitney U-test were used for statistical analysis of groups. Values of $P<0.05$ were considered significant and are listed in the figure legends (Prism 7, GraphPad, USA). The investigators were not blinded to the genotype of the mice/cells, but they were blinded to all sample and tissue analyses. Sample sizes were selected on the basis of preliminary results to ensure a power of 80% with 95% confidence between populations. All samples were analyzed in triplicate and were repeated at least three times, showing a representative result.
