# Supplementary material for: RAI16 maintains intestinal homeostasis and inhibits NLRP3‐dependent IL‐18/CXCL16‐induced colitis and the progression of colitis‐associated colorectal cancer
Source: Clin Transl Med. 2022 Aug 28;12(8):e993. doi: 10.1002/ctm2.993 (PMC9420419; doi:10.1002/ctm2.993)
Supplement: Supplementary file 2 — Supporting Material [file CTM2-12-e993-s002.pdf]

Supplementary figure legends

Figure S1. The model depicting the role of RAI16 in maintaining intestinal homeostasis and how loss of RAI16 promotes colitis and CAC.

RAI16 promotes IL-18 production in response to microbial DNA in colon. This is required for a balanced expression of antimicrobial peptides, including Reg3β and Reg3γ, to prevent dysbiosis. Conversely, loss of RAI16 impairs IL-18, Reg3β and Reg3γ production, resulting in *Prevotella* and *Bacteroides* dominated dysbiosis and heightened susceptibility to colitis or chronic colitis. RAI16 interacts with DynC2H1, regulating NLRP3 inflammasome dependent IL-18 production during DSS treatment. Loss of RAI16, the interaction of DynC2H1 and NLRP3 was enhanced, resulting NLRP3 inflammasome activation and increased IL-18 production, which induced exacerbated colitis. Increased IL-18 production induces CXCL16 production, which recruits immunosuppressive MDSC, and enhances tumor cell proliferation and migration during the resolution of colitis, which may eventually lead to colorectal cancer together.

Figure S2. RAI16 expression is decreased in IBD and CRC patients.

A. Box plot of RAI16 mRNA in healthy controls and IBD specimens (using dataset GSE9452 and GSE3365). In boxplots (middle line depicts the median and the whiskers the min-to-max range). **B.** RAI16 was downregulated in human CRC tissues. The relative mRNA level of RAI16 examined in human CRC-M0(n=459), CRC-M1 (n=73) and normal (n=789) tissues. **C.** Kaplan-Meier overall survival (OS) curves for CRC-M0 patients (n=459) and CRC-M1 patients (n=73). The data represent the mean ± S.E.M., and statistical significance was determined by a two-tailed Student's t-test unless otherwise indicated. ***$p < 0.001$.
