# Supplementary figures and images for: RAI16 maintains intestinal homeostasis and inhibits NLRP3‐dependent IL‐18/CXCL16‐induced colitis and the progression of colitis‐associated colorectal cancer
Source: Clin Transl Med. 2022 Aug 28;12(8):e993. doi: 10.1002/ctm2.993 (PMC9420419; doi:10.1002/ctm2.993)

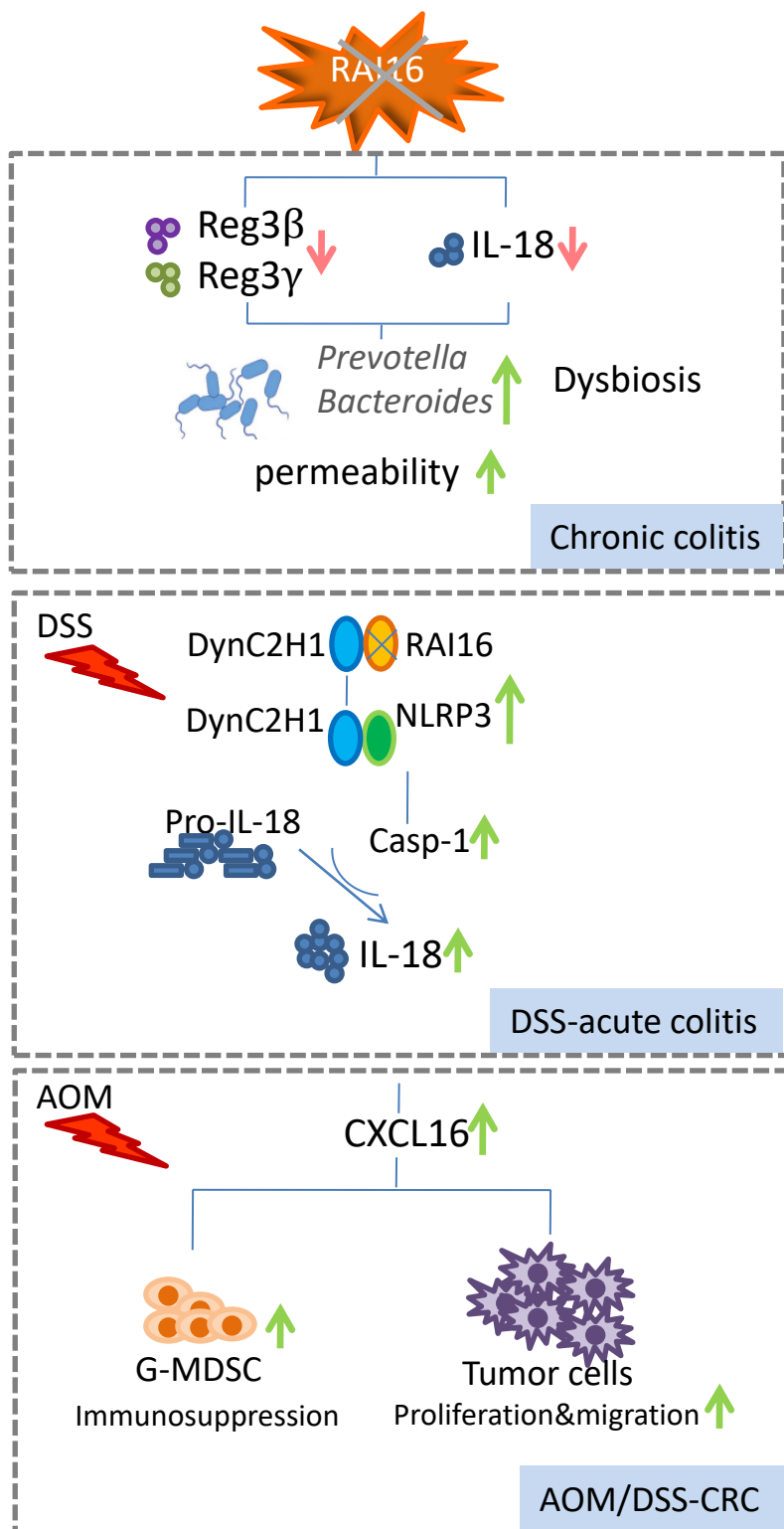

**Figure S1**

Supplement: Supplementary file 3 — Supporting Materiali [file CTM2-12-e993-s003.pdf]

**A**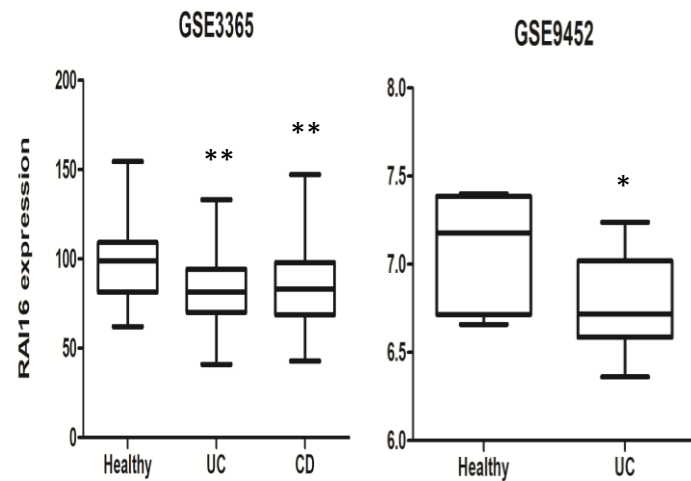**B**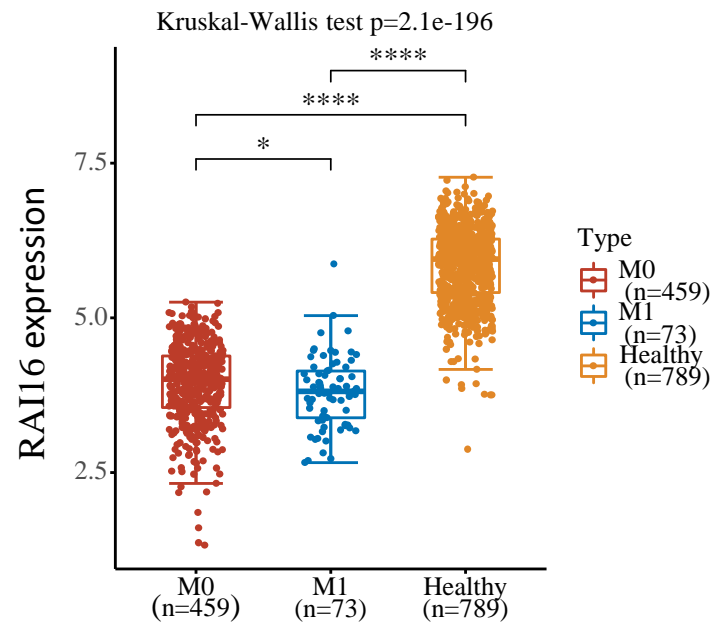**C**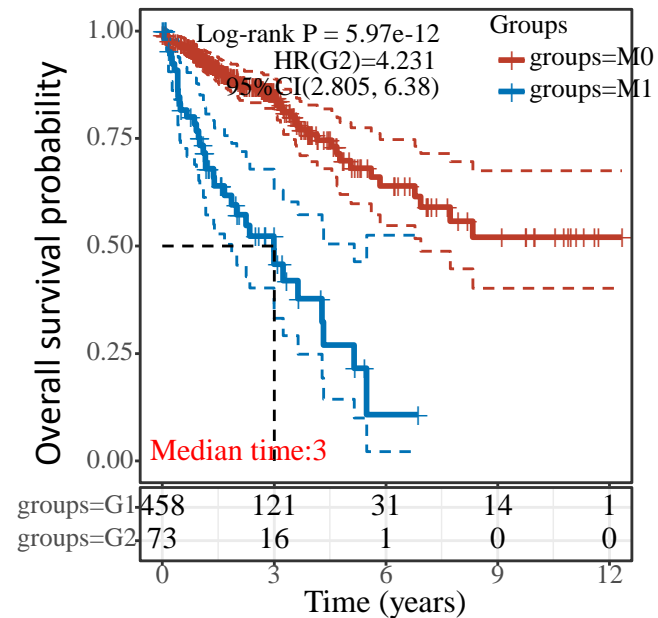**Figure S2**

Supplement: Supplementary file 4 — Supporting Material [file CTM2-12-e993-s004.pdf]
